# Considering the Role of Extracellular Matrix Molecules, in Particular Reelin, in Granule Cell Dispersion Related to Temporal Lobe Epilepsy

**DOI:** 10.3389/fcell.2022.917575

**Published:** 2022-06-06

**Authors:** Jennifer Leifeld, Eckart Förster, Gebhard Reiss, Mohammad I. K. Hamad

**Affiliations:** ^1^ Department of Neuroanatomy and Molecular Brain Research, Medical Faculty, Ruhr University Bochum, Bochum, Germany; ^2^ Department of Biochemistry I—Receptor Biochemistry, Faculty of Chemistry and Biochemistry, Ruhr University Bochum, Bochum, Germany; ^3^ Institute for Anatomy and Clinical Morphology, School of Medicine, Faculty of Health, Witten/ Herdecke University, Witten, Germany

**Keywords:** temporal lobe epilepsy, granule cell dispersion, reelin, febrile seizures, hippocampal sclerosis, extracellular matrix

## Abstract

The extracellular matrix (ECM) of the nervous system can be considered as a dynamically adaptable compartment between neuronal cells, in particular neurons and glial cells, that participates in physiological functions of the nervous system. It is mainly composed of carbohydrates and proteins that are secreted by the different kinds of cell types found in the nervous system, in particular neurons and glial cells, but also other cell types, such as pericytes of capillaries, ependymocytes and meningeal cells. ECM molecules participate in developmental processes, synaptic plasticity, neurodegeneration and regenerative processes. As an example, the ECM of the hippocampal formation is involved in degenerative and adaptive processes related to epilepsy. The role of various components of the ECM has been explored extensively. In particular, the ECM protein reelin, well known for orchestrating the formation of neuronal layer formation in the cerebral cortex, is also considered as a player involved in the occurrence of postnatal granule cell dispersion (GCD), a morphologically peculiar feature frequently observed in hippocampal tissue from epileptic patients. Possible causes and consequences of GCD have been studied in various *in vivo* and *in vitro* models. The present review discusses different interpretations of GCD and different views on the role of ECM protein reelin in the formation of this morphological peculiarity.

## Introduction

The organization of neuronal cell bodies and fiber projections in ordered layers represents a fundamental principle of the mammalian cerebral cortex and is essential for proper brain function. Disruption of this ordered layering is often associated with functional deficits or a decreased threshold for the occurrence of epileptic seizures, which happens to be the case in the reeler mouse mutant, which lacks expression of the protein reelin. Reelin is best known as a protein that is secreted by Cajal-Retzius (CR) cells into the ECM, and is essential for the correct positioning of migrating neurons.

Epilepsy is a neurological disorder associated with cerebral seizures characterized by chronic overexcitation of neurons in the brain and represents one of the most common neurological diseases worldwide. The most common form of anatomically classifiable focal epilepsy is temporal lobe epilepsy (TLE), where epileptic discharges originate in the temporal lobe (TL), frequently being pharmaco-resistant. TLE is sometimes associated with a history of febrile seizures (FS) and the so-called Ammon’s horn or hippocampal sclerosis (HS), characterized by a loss of hippocampal neurons. HS, in turn, is often observed in association with astrogliosis or microgliosis, central nervous system (CNS) conditions characterized by proliferation of astrocytes and microglia, respectively, suggestive of CNS injury and inflammation. TLE can be associated with GCD, which is mostly considered as a morphological malformation of the dentate gyrus (DG), reflecting a pathological condition of hippocampal granule cells (GCs) leading to disintegration of their dense layering, reminiscent of the morphological malformations seen in the reeler mutant mouse. GCD has been reported to be associated with reelin deficiency. While the role of reelin for GCD and the clinical picture of TLE is under debate, a recent study suggests that GCD may represent a variation within the normal range rather than a pathological abnormality.

## Temporal Lobe Epilepsy, Febrile Seizures, and Granule Cell Dispersion

It is estimated that epilepsy affects approximately 50 million people worldwide. Therefore, it can be considered as one of the most common neurological disorders (Dua et al., 2006; Guerreiro, 2016; Falco-Walter, 2020). A meta-analytic study of the prevalence (the number of existing cases) and incidence (new-onset cases) of epilepsy worldwide included a total of 222 studies (197 on prevalence and 48 on incidence) (Fiest et al., 2017). This study identified a prevalence of active epilepsy of 6.38 per 1,000 persons and a lifetime prevalence of 7.60 per 1,000 persons, which did not differ with respect to age or sex (Fiest et al., 2017). The annual cumulative incidence of epilepsy was found to be 67.77 per 100,000 persons (Fiest et al., 2017).

**FIGURE 1:**
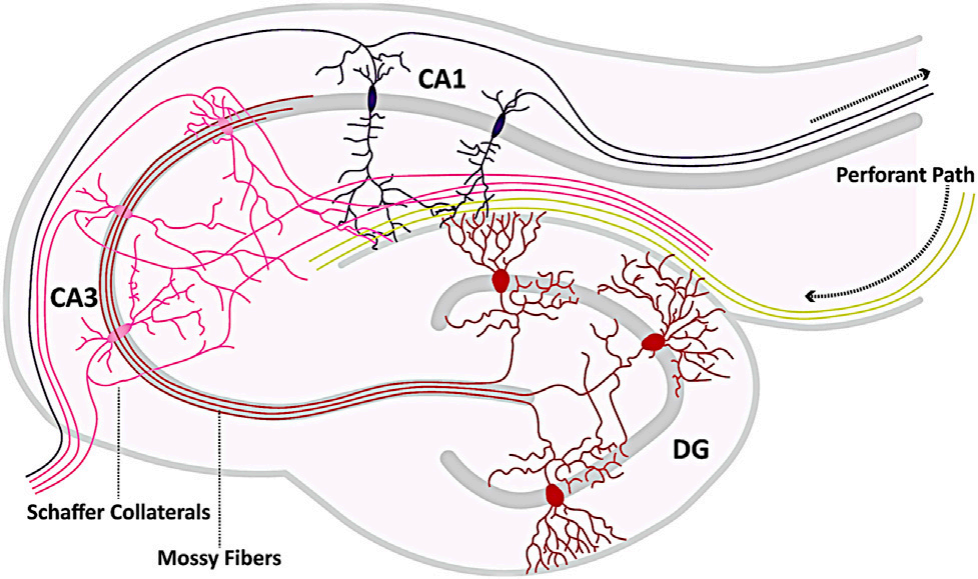
Hippocampal circuitry. Shown is a schematic representation of the hippocampal circuitry (tri-synaptic circuit). The hippocampus receives afferents from the neighboring entorhinal cortex, which enter the hippocampal formation primarily *via* the Perforant Path (yellow) and form synaptic contacts with both pyramidal cells (pink, purple) and partially with granule cells (GCs, red). The axons of the GCs (mossy fibers) innervate the pyramidal cells of the CA3 region (pink), from whose axons the Schaffer collaterals branch to form synapses with the dendrites of the CA1 pyramidal cells (purple). Axons of the pyramidal cells (pink, purple) eventually exit the hippocampus as efferent elements. The template for this figure was (Marín-Padilla, 1998; Twible et al., 2021).

TLE is the most common form of epilepsy with focal seizures, characterized by recurrent, spontaneous seizures originating in the TL of the brain (Pascual, 2007; Nayak and Bandyopadhyay, 2021). The ILAE distinguishes two main types of TLE: mesial TLE (MTLE) and lateral TLE (LTLE) (Panayiotopoulos, 2005; Berg et al., 2010). The latter arises in the lateral part of the TL, meaning the lateral surface adjacent to the neocortex (Kutsy, 1999; Kim et al., 2003; O’muircheartaigh and Richardson, 2012). MTLE, on the other hand, originates in the MTL, i.e., in the hippocampus and nearby structures, such as the amygdala and the entorhinal cortex, and in structures surrounding it, such as the parahippocampal gyrus (Engel, 2001; Pascual, 2007; Nayak and Bandyopadhyay, 2021). At least 80% of all TLE cases originate in the hippocampus due to neuronal overexcitability (Tatum, 2012) making it one of the most susceptible areas for TLE and MTLE, respectively. Figure 1 depicts an overview of the hippocampus and the tri-synaptic circuit.

HS and FS are associated primarily with TLE because retrospective studies or Magnetic Resonance Imaging (MRI) studies repeatedly found that many patients who underwent surgery for intractable TLE had FS in childhood and showed signs of HS. It has been speculated that FS lead to structural damages in the TL, as a result of which HS develops, eventually terminating in TLE (Mathern et al., 1997; Vanlandingham et al., 1998; Cendes, 2004). Another theory is that innate hippocampal abnormalities predispose the TL to be particularly susceptible to prolonged infantile FS, eventually leading to TLE (Annegers et al., 1987; Vanlandingham et al., 1998; Blümcke et al., 2002; Cendes, 2004). However, looking at the available literature, it is striking that there is no clarity regarding this relationship (Shinnar, 1998; Sloviter and Pedley, 1998; Kälviäinen and Salmenperä, 2002; Cendes, 2004; Blümcke et al., 2009). Thus, it is still controversial whether persistent FS lead to HS or MTLE. Retrospective studies performed by MRI or postoperative histopathology have reported many cases of adults with intractable MTLE had prolonged FS in childhood (Abou-Khalil et al., 1993; Cendes et al., 1993; French et al., 1993; Kuks et al., 1993; Trenerry et al., 1993; Maher and Mclachlan, 1995). Although HS is particularly associated with MTLE, it has also been observed *post-mortem* (PM) associated with other forms of epilepsy (Thom, 2014). The incidence for HS in PM series related to all forms of epilepsy is 30.5%–45% and is 56% related to MTLE (Novy et al., 2013; Thom, 2014). In a study with a total of 3,311 TLE patients, HS was detected in 48% (Blümcke, 2009; Blümcke et al., 2012). In another study, conducted on a total of 4,512 epilepsy patients in whom the reasons for surgical resection varied, HS was detected in 35.2% (Blümcke et al., 2002). Nevertheless, population-based and prospective studies have not necessarily been able to establish this relationship (Nelson and Ellenberg, 1976; Lee et al., 1981; Janszky et al., 2003; Tarkka et al., 2003). Limitations of retrospective studies include, for example, certainty about the exact diagnosis as well as the exact characteristics of the previous FS (Camfield et al., 1994). In addition, it is possible that patients had prior pathologies that led to FS or that these two conditions are simply not correlated (Tarkka et al., 2003; Thom, 2014). Moreover, retrospective studies usually examine only highly selected patients with drug-resistant MTLE (Tarkka et al., 2003; Cendes, 2004) with an epileptic seizure duration of an average of 23 years (Blümcke, 2009), and epilepsy-free controls are usually absent (or it is not stated) because control sections are often not available (Abou-Khalil et al., 1993; Blümcke et al., 2002). For example, in one study (Cendes et al., 1993), a total of 43 TLE patients (with LTLE and MTLE) who underwent temporal lobectomy were examined by MRI, and the sections were subsequently examined for HS. Of these, 25% had severe, 42% moderate, and 33% mild HS (Cendes et al., 1993). Prolonged FS was previously suffered by 35% of the patients (Cendes et al., 1993). Because no sections from the control group were available, they could not be examined for HS, but the authors found by means of MRI that the patients suffering from TLE had significant smaller volumes of amygdala and hippocampus compared to the controls (Cendes et al., 1993). In another study, a total of 32 adolescent patients with previously prolonged FS or an unprovoked FS after the first seizure was examined using MRI by a radiologist blinded to the patients’ history compared with 32 control patients selected for age, sex, and handedness who had previously had only a simple FS without a subsequent seizure, and none of the patients were found to show HS in the MTL, nor did hippocampal volume differ between the two groups (Tarkka et al., 2003). Drawbacks of this study are certainly the low number of (only adolescent) patients studied, with most temporal lobectomies being performed in older patients with more advanced MTLE (Blümcke et al., 2009). For a more detailed critical discussion of this study, see (Scott et al., 2003). In a population-based study examining the frequency of developing epilepsy after FS, 2% of 1,706 children studied who had suffered at least one FS and were followed until 7 years of age developed epilepsy, whereas children whose neurologic or developmental status was suspicious or abnormal before a seizure and whose first seizure was complex (i.e., >15 min, multiple, or focal seizures) were 18 times more likely than children without FS to develop epilepsy (Nelson and Ellenberg, 1976). Previously unremarkable children with a noncomplex first FS, developed epilepsy in 1.1% of cases, which was higher than in children without FS, so it was concluded that the children’s previous neurological and developmental status and characteristics of the first FS were important predictors of epilepsy after FS (Nelson and Ellenberg, 1976). In another retrospective study conducted on the resections of 243 TLE patients performed for intractable seizures, 32% of all patients had prehistoric FS, of whom, in turn, 52% also had HS, the latter observed in a total of 25% of all patients, suggesting a stronger association between FS and HS, but leading the authors to conclude that HS cannot be considered the exclusive cause of TLE, as HS was observed in most cases in association with other pathologies (Tassi et al., 2009).

In addition to FS, regarding the patient’s medical histories, there are a number of other putative risk factors that are commonly found in TLE patients including birth trauma, head injury, and meningitis (Blümcke et al., 2002; Wieser, 2004). Moreover, HS is not exclusively found in TLE patients, but can also be observed in other diseases, e.g., Alzheimer’s Disease (AD), Dementia with Lewy bodies (DLB), or can occur along with brain abnormalities such as white matter hyperintensities and is also frequently observed in the elderly associated with anoxic or ischemic injury (Zarow et al., 2008; Kotrotsou et al., 2015; Mak et al., 2016; Fiford et al., 2017; Yu et al., 2020). HS also appears to vary with age. For instance, in a study (Blumcke et al., 2017) of brain resections from 9,523 surgery-treated patients due to drug-resistant epilepsy, with the TL being involved in 71.9% of all cases, HS was diagnosed in 44.5% of adults and in 15% of children.

It should also be noted that MTLE may also be genetically predisposed, which is referred to as familial MTLE (Cendes, 2004). Thus, MRI abnormalities suggestive of developmental abnormalities of the medial temporal structures or hippocampus have been noted in unaffected and affected individuals with familial MTLE, some of whom have an appearance distinct from nonfamilial TLE (Depondt et al., 2002; Kobayashi et al., 2002; Kobayashi et al., 2003; Andrade-Valença et al., 2008; Crompton et al., 2010). Investigations of familial MTLE showed that MRI evidence of HS was not necessarily related to seizure severity and occurred even in individuals who never had a seizure (Kobayashi et al., 2002). Different possible gene loci for familial MTLE have been described previously, indicating complexity and genetic heterogeneity in familial focal epilepsy (Hedera et al., 2007; Chahine et al., 2013; Kasperaviciute et al., 2013; Sheilabi et al., 2020). For example, a meta-analysis revealed a genome-wide significant association for MTLE with HS in the α1-subunit of the voltage-gated sodium channel (VGSC) (Kasperaviciute et al., 2013) and further experiments have demonstrated a reduction in the expression profile of the β4-subunit of the VGSC at both the transcriptional and translational levels in sclerotic hippocampal tissue from patients with pharmaco-resistant MTLE (Sheilabi et al., 2020). Because the β4-subunit plays an important role in determining the gating of the VGSC, it has been suggested that mutation of the sodium channel subunit may not only alter channel gating possibly resulting in epileptic neuronal activity, but may also affect the binding sensitivities of pharmaceuticals, as they often act in a use-dependent manner (Sheilabi et al., 2020). In summary, the numbers with respect to TLE related phenomena like FS and HS vary in the existing studies conducted on this topic.

The different findings reported in the literature also result from the fact that the diagnosis of HS has not always been uniformly classified, which is why the ILAE agreed in 2017 to adopt new definitions for this pathology in TLE, with the main histopathological feature of TLE-associated HS being the segmental loss of pyramidal neurons, which can occur in every area of the Ammon’s horn (*Cornu Ammonis*, CA) (Blümcke et al., 2012; Blümcke et al., 2013; Nayak and Bandyopadhyay, 2021). According to the ILAE’s new definition, HS type 1 is defined as the severe segmental loss of pyramidal neurons in the CA1 and CA4 areas (Blümcke et al., 2013; Thom, 2014; McIntosh and Joe, 2021), type 2 features major cell loss in the CA1 region, and HS ILAE type 3 in the CA4 area (Blümcke et al., 2013; Thom, 2014; McIntosh and Joe, 2021). In addition, all three types display gliosis (Blümcke et al., 2013). HS is always associated with astrogliosis and is traditionally referred to as “Ammon’s horn sclerosis” (Sommer, 1880; Blümcke et al., 2012). Astrogliosis denotes the proliferation of reactive astrocytes in response to CNS injury and, as a result, the affected tissue hardens (Sofroniew and Vinters, 2010; Nayak and Bandyopadhyay, 2021). This CNS condition can exhibit a wide spectrum of alterations, from reversible cell hypertrophy with tissue preservation to persistent scarring accompanied by tissue remodeling (Blümcke et al., 2013; McIntosh and Joe, 2021).

Some neuroimaging studies of patients with TLE also showed that HS is not always localized (Bronen et al., 1995; Woermann et al., 1998), although this has not been confirmed in all studies (Vos et al., 2020). In a neuropathological study of the extent of HS in epilepsy along the longitudinal axis of the hippocampus, ten autopsies from patients with a long history of drug-refractory epilepsy and from three control subjects were systematically examined at seven coronal anatomical levels along the body to the tail (Thom et al., 2012). In this study, the pattern of sclerosis was the same at all levels in less than one-third of cases and showed marked longitudinal variability in most cases, leading the authors to suggest that studies of HS in sections derived exclusively from one coronal level cannot necessarily be considered fully representative of the pathology (Thom et al., 2012).

**FIGURE 2:**
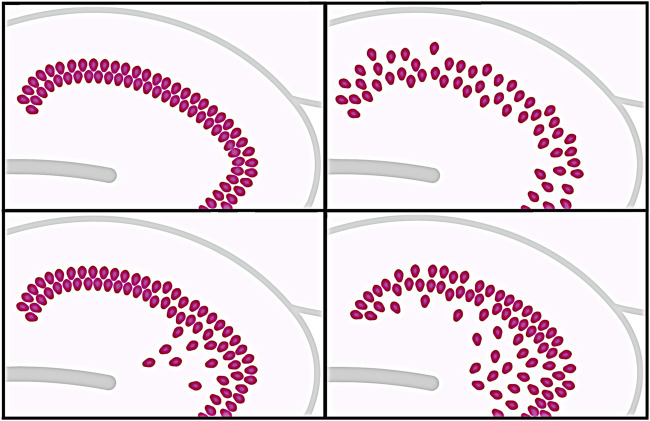
Different patterns of granule cell dispersion (GCD). The different possible patterns of GCD are illustrated. A granule cell layer (GCL) considered normal in the classical sense represents a compact band of cells with a relatively high cell density and is clearly delineated against the molecular layer (ML) (top left). In the disaggregated form of GCD, the GCL appears less densely packed and the boundary against the ML is blurred (top right). Ectopic granule cells, some of which can be found deep within the hilar region (bottom left), are shown in comparison to developmental GCD (bottom right), in which young differentiated GCs migrate from the hilar region to the GCL to ultimately build a compact GCL.

In addition to early-onset FS and HS, TLE is frequently associated with the putative pathogenic appearance of GCD, in which the GCs of the dentate gyrus (DG) abandon their “normally” dense layer and subsequently appear in the ML, resulting in an expansion of the GCL sometimes showing a bi-laminated pattern and in the loss of a clear boundary between the two cell layers (Houser, 1990; Lurton et al., 1997; Thom et al., 2005b; Gong et al., 2007; Koyama et al., 2012) (see Figure 2 for overview of possible GCD patterns). Yet, GCD has often been identified as a hallmark in the hippocampus not only in epilepsy patients, but also in sudden unexplained deaths in infants or children and in numerous animal models that are frequently used to study epilepsy (Koyama et al., 2012; Chai et al., 2014; Kinney et al., 2015; Hefti et al., 2016).

Two hypotheses exist for the occurrence of GCD. First, GCD could reflect a migratory defect during GC neurogenesis that persists into adulthood (Altman and Das, 1966; Eriksson et al., 1998; Kempermann et al., 1998), associated with early precipitating injuries before the age of 4 years, particularly FS, i.e., an extrinsic insult (Houser, 1990; Lurton et al., 1997). The second hypothesis assumes an intrinsic failure, such as a defect in the genetically determined developmental program (Houser, 1990; Harding and Thom, 2001). In contrast, other studies suggest that GCD is not a common consequence of recurrent seizures at a young age, but rather depends on the type of epileptic syndrome (Mathern et al., 1997). To date, this question has not been clearly confirmed or disproved, and now another hypothesis has been added, according to which GCD does not represent a pathological change at all, but is rather a natural non-specific variation (Roy et al., 2020). In this publication, GCD, previously commonly considered pathogenic, is described as an “erroneous dogma” (Roy et al., 2020). The authors (Roy et al., 2020) retrospectively examined 147 pediatric human hippocampi, including a total of 21 cases with epilepsy and 126 controls, and identical morphologic spectra of GCD were obtained in normal and seizure-affected brains. In addition, sections through the entire antero-posterior axis of a control hippocampus were examined, and the repeated occurrence of different morphologies of the GCL, that is, compact, focally disaggregated, and bilaminar morphology was observed (Roy et al., 2020) (Figure 2). Therefore, the authors conclude that the scatter of GCs represents a normal variation instead of a specific trait of epilepsy and suggest that sampling biases are responsible for a false dogma (Roy et al., 2020). In fact, many studies, especially those performed on human resected specimens, included few and sometimes no (appropriate) controls. For an overview of relevant studies, see (Roy et al., 2020). The number of controls examined from specimens of patients without epilepsy ranged from two to a maximum of eight in most studies (Houser, 1990; Lurton et al., 1997; El Bahh et al., 1999; Harding and Thom, 2001; Thom et al., 2002; Thom et al., 2005a; Blümcke et al., 2007; Abrahám et al., 2011); a total of four studies in which GCD was identified as a specific characteristic of epilepsy included no controls at all (Blümcke et al., 2009; Bae et al., 2010; Marucci et al., 2010; Freiman et al., 2011). In contrast, the number of examined specimens of epilepsy patients in the mentioned studies varies between ten (Freiman et al., 2011) and 206 (Thom et al., 2002) and is thus significantly higher than the number of included controls. A more recent study also found an association between GCD and TLE, particularly in the presence of HS, and the occurrence of GCD was additionally associated with an initial precipitating injury as well as with a higher number of epileptic focal seizures (Jardim et al., 2021). In the aforementioned study (Jardim et al., 2021), specimens from 108 TLE patients with unilateral HS were examined for histopathologic hippocampal changes compared with specimens from twelve individuals without epilepsy or other neurologic conditions, also significantly outnumbering the tested epilepsy specimens. In addition, there are two other studies not mentioned in the previously described publication (Roy et al., 2020) that also found a correlation between GCD and TLE (Duarte et al., 2018; Jeong et al., 2018). These found an increased probability of abnormal distribution of GCs in the DG in patients with longer duration of epilepsy, with 77 cases of epilepsy compared to twelve controls included in the study (Duarte et al., 2018). In the other study, quantification of the width of the GCL of patients diagnosed with TLE-HS (eight), normalized to the width in normal controls without HS (four), a significant increase in GCL width was observed (Jeong et al., 2018). In addition to the report from (Roy et al., 2020), only one further exists in which GCD was found in two neurologically normal pediatric patients, from which it was concluded that the appearance of GCD may not be exclusively related to epilepsy and may be a separate developmental disorder (Harding and Thom, 2001).

**FIGURE 3:**
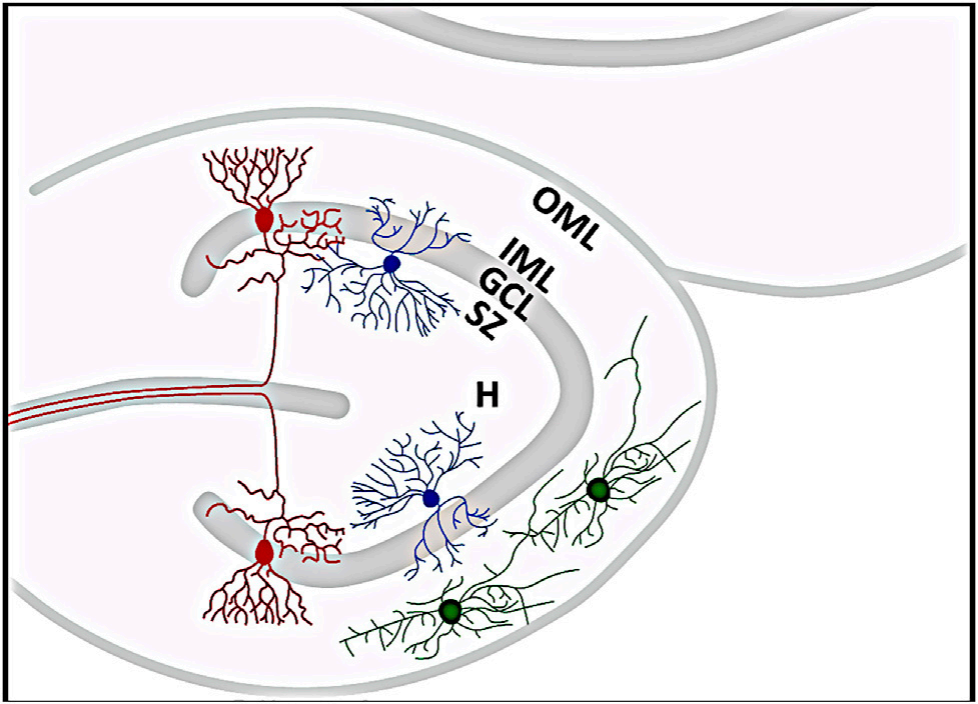
Histological organization of the dentate gyrus (DG). Red neurons represent granule cells whose somata form the granule cell layer (GCL). Just below is the subgranular zone (SZ), where reelin-expressing interneurons (blue) can be found, and above is the molecular layer, which can be further subdivided into an inner (IML) and an outer molecular layer (OML). The outer OML harbors reelin-synthesizing Cajal-Retzius cells (green). The hilar area (H) of the DG is located below the SZ. The template for this figure was (Marín-Padilla, 1998; Twible et al., 2021).

Considering the number of included controls compared to the amount of examined specimens of TLE patients in the performed studies, it might indeed be possible that sampling bias could be responsible for the association between GCD and TLE. But there are other possible causes that could have contributed to the different findings. One difference from many other studies is that the study (Roy et al., 2020) examined mainly pediatric specimens. During development, early generated GCs migrate from the hilus of the DG towards the marginal zone (MZ) to their final positions (Förster et al., 2006; Hayashi et al., 2015), so a transient distribution of newborn GCs in the hilus could reflect a natural stage of early postnatal development of the DG, which, conversely, could be rapidly mistaken for pathological GCD (Weninger et al., 2021). Figure 3 illustrates the histological organization of the DG. It has to be admitted that cell migration into the GCL occurs during the first eight postnatal months, when immature cells gradually disappear from the subgranular zone near the hilus (Seress, 1992; Seress et al., 2001). However, statistical analyses revealed no significant correlation between the occurrence of GCD and age at death, cell loss, PM interval, sex, clinical diagnosis, seizure, or other clinical history between control as well as seizure brains (Roy et al., 2020). Notably, gliosis was observed more frequently in some of the hippocampi affected by seizures but was independent of GCD, although the DG did not show increased GFAP-positive astrocytes in all epilepsy cases (Roy et al., 2020). Moreover, in contrast to the determination of HS, there is currently no consensus on the criteria for determining GCD, which depend in part on the type of measurement and range from complex morphometric analyses to subjective assessment of DG histology, what makes standardized identification and assessment of GCD difficult (Lurton et al., 1997; Mathern et al., 1997; Haas et al., 2002; Blümcke et al., 2009; Abrahám et al., 2011). Another discrepancy important to note is that there are significant differences between the aforementioned studies and the one performed by (Roy et al., 2020) in terms of the hippocampal specimens studied. For instance, all studies were limited to biopsies from (mostly adult) patients with intractable TLE and a usually long history of epileptic seizures (Houser, 1990; Lurton et al., 1997; El Bahh et al., 1999; Thom et al., 2002; Thom et al., 2005a; Blümcke et al., 2007; Blümcke et al., 2009; Bae et al., 2010; Marucci et al., 2010; Abrahám et al., 2011; Freiman et al., 2011; Duarte et al., 2018; Jeong et al., 2018; Jardim et al., 2021). Patients whose tissues were examined had an average duration of epilepsy of at least 17.5 years (Lurton et al., 1997) to a maximum of 24.8 years (Thom et al., 2002). In contrast, the average duration of epileptic seizures in the study conducted by (Roy et al., 2020) was only three years. Moreover, only the hippocampi of cadaveric epilepsy cases and controls, respectively, were examined (Roy et al., 2020). This also applies to the study performed by (Harding and Thom, 2001), who also found GCD in control hippocampi of pediatric cadaveric patients, and again the duration of epileptic seizures in the one epileptic patient examined lasted only about six months (Harding and Thom, 2001). These serious differences may well have affected the results of the different studies.

Investigating GCD as a cause or consequence of epilepsy is particularly difficult in surgical series on human specimens, as these typically involve resections of selected patients, most of whom have had preexisting hippocampal damage detected by MRI with an advanced disease stage often underlying a strong heterogeneity in terms of potentially epileptogenic injuries (Harding and Thom, 2001; Fisher et al., 2014; Jeong et al., 2018; Jardim et al., 2021). In parallel with the apparent lack of a suitable number of non-epileptic, age-matched human control brain samples, the study of epileptogenesis on human brain tissue is particularly limited by statistically valid numbers of homogeneous sample series in terms of clinical, pathological, and individual parameters, which is why research largely relies on animal models herein (Jimenez-Mateos et al., 2012; Dębski et al., 2016; Becker, 2018).

## Animal Models Mirroring Human Temporal Lobe Epilepsy

Animal models but also *in vitro* models derived from animal tissue have been used in an attempt to at least partially compensate for the lack of human control hippocampi and to gain insight into early stages of epileptogenesis, for which human hippocampal tissue is usually unavailable (Becker, 2018; Nirwan et al., 2018). Moreover, animal models play an important role in drug discovery and development, and molecular changes observed in both focal epileptic lesions in humans and in corresponding animal models should be more likely to be of pathogenetic significance (Becker, 2018; Nirwan et al., 2018). In the most commonly used animal models, rodents, primarily mice and rats, are treated with chemoconvulsants, with kainate (KA) and pilocarpine (PL) being the most commonly used, inducing recurrent chronic seizures in treated animals that reflect important neuropathological changes of human TLE (Leite et al., 2002; Becker, 2018). In this regard, systemic or local administration of chemoconvulsants in rodents results in severe depolarization of neurons and subsequent epileptic seizures, with the focus of damage in the hippocampal formation (Sharma et al., 2007; Becker, 2018). Although many animal models can be used to reproduce the pathophysiological changes such as HS, GCD and spontaneous recurrent seizures, no single animal model shares all characteristics of TLE (Nirwan et al., 2018). For example, various parameters, such as sex, age, and weight, influence the sensitivity to KA (Nirwan et al., 2018). Intrahippocampal injection of KA or PL results in bilateral electrical spikes and after-discharges as well as behavioral seizures in rodents comparable to *status epilepticus* (SE) and subsequent chronic recurrent spontaneous seizures (Becker, 2018; Nirwan et al., 2018; Freiman et al., 2021; Moura et al., 2021). In chemoconvulsant-treated rodents, GCD also develops (Freiman et al., 2021; Moura et al., 2021), which may indicate that it is a concomitant of TLE rather than a natural variation. However, in research comparing KA- versus PL-injected animals, GCD was observed exclusively in KA-treated animals, but not in those treated with PL (Moura et al., 2021). This finding rather suggests that KA has an additional local effect with epileptic activity alone not being sufficient to cause GCD (Moura et al., 2021), especially since epileptic neuronal activity after KA treatment has also been observed on the side contralateral to the injection site (Moura et al., 2021). In contrast, previous studies have shown that the width of the GCL was significantly greater after PL treatment compared with untreated control animals, but exclusively in animals in which PL treatment induced SE (Mello et al., 1992; Mello et al., 1993) speaking against the conclusion that epileptic discharges and SE, respectively, are not sufficient to evoke GCD. In another model, animals are implanted unilaterally or bilaterally with two stimulating and two recording electrodes in the perforant pathway (Figure 1) and then electrically stimulated to induce seizures (Bumanglag and Sloviter, 2008; Nirwan et al., 2018). Although GCD is not subsequently manifested, degeneration of DG cells is observed after stimulation (Sloviter et al., 1996). In a study in which epileptic seizures were induced in rats using amygdala kindling, more ectopically located GCs were found in treated animals, but no change was observed between controls and electrically stimulated animals in terms of GCL volume or width (Fournier et al., 2010). Interestingly, another study found an increase in the width of the GCL one day after the last seizure triggered by amygdala kindling, and this change had regressed in animals examined one month later (Singh et al., 2013). In contrast, other morphological features often observed in other models of TLE, such as ectopic GCs, were not observed in kindled animals examined at either time point (Singh et al., 2013). In another animal model of experimental TLE, FS are triggered in rodents to resemble the state of human FS (Jiang et al., 1999; Bender et al., 2004; Koyama and Matsuki, 2010; Koyama et al., 2012). For this purpose, animals are kept hyperthermic at 39°C–41.5°C for 30 min, resulting in seizures lasting approximately 20 min (Bender et al., 2004). Similarities between experimentally induced hyperthermic seizures in rats and TLE in humans have been reported previously (Jiang et al., 1999). Using the described model, it was examined whether FS can induce GCD, finding that convulsive rats indeed displayed ectopic GCs in the hippocampus, but this was more likely triggered by the seizures themselves, and temperature elevation alone was not sufficient to evoke GCD (Koyama et al., 2012). The authors further demonstrated that abnormal migration of neonatal-generated GCs leads to GC ectopy, persisting into adulthood (Koyama et al., 2012), whereas another study concluded that fully differentiated GCs become motile after KA-induced epileptiform activity, resulting in the manifestation of GCD (Chai et al., 2014). In this regard, decreased expression of the ECM protein reelin, which is illuminated in more detail in the next section, was found after application of KA, both at the mRNA and protein levels, with interneurons in the hilus and the subgranular zone being particularly affected (Chai et al., 2014) (Figure 3). Results from studies using animal models have to be taken with caution as it is questionable to which extend, they reflect the respective human disease state and the findings may vary depending on the model used. Nevertheless, they are important to gain insight into pathological mechanisms underlying human TLE and drug design for which human tissue is not available. However, it is striking that GCD, unlike in human hippocampi, is not observed in the healthy rodent hippocampus. It is therefore conceivable that this phenomenon is a natural variation in humans but not in rodents.

## Extracellular Matrix and the Extracellular Matrix Molecule Reelin

The ECM constitutes a three-dimensional, cell-free matrix surrounding neurons and glial cells in the CNS, which synthesize and secrete ECM molecules (Dityatev, 2010; Lau et al., 2013; Lillis, 2021). The ECM is rich in proteoglycans and hydrophilic glycosaminoglycans and also contains collagen, elastin, fibronectin, laminin, and other glycoproteins and accounts for around 20% of cerebral volume (Cragg, 1979; Lau et al., 2013; Theocharis et al., 2016). The structure of the ECM of the CNS is heterogeneous and depends on various factors, e.g., on the present cell types (Dityatev and Fellin, 2008). For example, the perineuronal network, which surrounds cell somata and proximal dendrites as well as at initial segments of axons of certain neurons and surrounds mainly parvalbumin-expressing GABAergic interneurons in the cerebral cortex and hippocampus, represents one form of ECM (Dityatev, 2010). Depending on its composition, this complex and diverse structure filling the extracellular space influences the structural plasticity of the tissue as well as cell-cell and cell-matrix interactions with a multitude of secreted growth factors (Dityatev, 2010). Thus, the ECM of the CNS forms a dynamic system and the constant adaptation of ECM structure to required conditions by regulating the expression, respectively the activity of extracellular proteases, is fundamental for the correct function of physiological processes (Ulbrich et al., 2021). Extracellular proteases include the plasmin system, the proteins ADAM (a disintegrin and metalloproteinase) and ADAMTS (ADAM with thrombospondin motifs), as well as matrix metalloproteases (MMPs) (Stevenson and Lawrence, 2018; Ulbrich et al., 2021). Besides degrading proteins, the latter is also capable of processing signaling molecules and in this way controls cellular signaling events (Rodríguez et al., 2010). The activity of MMPs is in turn regulated by tissue inhibitor of metalloproteinases (TIMPs) (Acar et al., 2015) and a disbalance between expression levels of MMPs and TIMPs has been associated with several pathologic conditions (Mizoguchi and Yamada, 2013). ECM molecules transmit signals by binding to cell surface receptors (Theocharis et al., 2016) and thereby influence higher-level processes, such as synaptic plasticity and brain development, the latter of which the ECM molecule reelin is particularly known to be responsible for (Dityatev et al., 2010).

Thus, the ECM is a fundamental component of neuronal processes, along with neurons and glial cells. Dysregulation of the ECM is associated with neurological diseases, including epilepsy (Dityatev, 2010). Injuries to the CNS, such as seizures, may activate the immune system, possibly leading to alteration of perivascular and perineuronal ECM, often accompanied by high expression and associated activity of matrix-remodeling extracellular proteases such as MMPs (Ulbrich et al., 2021). Activation of astrocytes enhances synthesis of ECM molecules and, together with increased expression of TIMP proteins, elicits aggregation of ECM molecules and activates signaling cascades involving various molecules such as integrins, Toll-like receptors, cell adhesion molecules, and ion channels (Ulbrich et al., 2021). Epileptic conditions in particular lead to aberrant expression of extracellular proteases, which in turn is associated with neurodegeneration, neuroinflammation, and concomitant altered synaptic plasticity (Konopacki et al., 2007; Lukasiuk et al., 2011; Mizoguchi and Yamada, 2013). The importance of the ECM is exemplified by hyaluronic acid (HA), a fundamental component of the ground substance of the ECM. Thus, enzymatic digestion of HA, by hyaluronidase is sufficient to trigger epileptic activity *in vitro* and *in vivo* (Vedunova et al., 2013; Balashova et al., 2019). Moreover, hyaluronan synthase ko mice exhibit spontaneous epileptic seizures (Arranz et al., 2014). Based on studies performed on animal models as well as on human tissue remnants, with respect to MMPs, MMP-9 is particularly associated with epilepsy, with epileptic activity leading to upregulation of MMP-9 (Szklarczyk et al., 2002; Konopacki et al., 2007; Wilczynski et al., 2008; Takács et al., 2010; Li et al., 2012; Acar et al., 2015; Pijet et al., 2018). In addition to HA and MMP-9, epileptic conditions lead to increased expression of other ECM molecules, such as tenascin R, tenascin C, and neuronal pentraxin 2, whereas other molecules, such as protein tyrosine phosphatase receptor type Z1 (phosphacan) are downregulated (Dityatev, 2010). Tenascin R molecules are, among others, present in perineuronal networks and, in di- or trimeric form, cross-link lecticans and thus have an ECM-stabilizing effect (Morawski et al., 2014). Tenascin R-deficient mice, although not epileptic, exhibit decreased perisomatic GABAergic inhibition and synaptic plasticity and show significantly increased neuronal activity in the CA1 region after PL treatment compared to wild-type mice (Saghatelyan et al., 2001; Brenneke et al., 2004; Pitkänen et al., 2014). These findings highlight the strong effect the ECM exerts on peripheral cells. Due to the importance of the ECM for proper CNS function, compounds modulating the effects of specific ECM molecules could serve as therapeutic interventions in various neurological conditions. For example, treatment with the MMP-9 inhibitor IPR-179 reduced the severity of seizures in the kindling model and reduced the number of spontaneous seizures in the KA model, with no side effects noted, and furthermore improved cognitive performance of treated rodents (Broekaart et al., 2021). Another MMP inhibitor that has been tested using the KA model is marimastat, also showing beneficial effects on seizure duration (Pijet et al., 2020). Another molecule that has already been studied intensively is the ECM molecule reelin.

Reelin is a large secreted ECM glycoprotein regulating many important processes in mammalian brain development, and dysregulation of reelin signaling has been linked to several brain diseases such as autism, schizophrenia, depression, AD, and, in particular, epilepsy (Ishii et al., 2016; Hirota and Nakajima, 2017; Santana and Marzolo, 2017; Wasser and Herz, 2017; Armstrong et al., 2019; Okugawa et al., 2020; Orcinha et al., 2021). The highly conserved human reelin gene is located on chromosome 7q22 and is *circa* 450 kbp long (Desilva et al., 1997; Manoharan et al., 2015). Composed of 3,461 amino acids, the reelin protein has a relative molecular mass of 388 kDa, and of 450 kDa in its glycosylated state (D’Arcangelo et al., 1995; Desilva et al., 1997; Jossin, 2020). With respect to its primary structure, reelin is subdivided into several major domains. An N-terminal signal peptide is followed by an F-spondin homology domain, which is joined by a unique region that displays no sequence similarity to previously known domains, featuring a series of reelin-specific repeats (Ichihara et al., 2001; Yasui et al., 2007; Manoharan et al., 2015). The C-terminal domain contains predominantly basic amino acids (D’Arcangelo et al., 1995; Jossin, 2020). Reelin is functional as a multimer and linkage is formed by disulfide bonds and by non-covalent interactions (Kubo et al., 2002; Yasui et al., 2011; Manoharan et al., 2015). After secretion into the ECM, reelin is proteolytically cleaved into smaller isoforms (Lambert de Rouvroit et al., 1999; Jossin et al., 2004), which may be important for the physiological function of the protein (Tinnes et al., 2011; Tinnes et al., 2013; Orcinha et al., 2016). In this process, reelin can be cleaved into different fragments, depending on which protease is active, although it is controversial which of the resulting reelin fragments is critical for the activation of the reelin signaling pathway (Lambert de Rouvroit et al., 1999; Lugli et al., 2003; Tinnes et al., 2013). Thus, some studies show that the N-terminal fragment is important for its biological function (Nakajima et al., 1997; Kubo et al., 2002; Orcinha et al., 2021), other studies focus on the C-terminal part (Nakano et al., 2007; Kohno et al., 2015) of the protein, while others suggest that a dimeric central fragment is signaling competent (Jossin et al., 2004; Yasui et al., 2007; Orcinha et al., 2021; Turk et al., 2021). Results of a recent study suggest an essential role for the dimeric central reelin fragment, but not the other reelin fragments or the monomeric central fragment, in the lipoprotein receptor-dependent activation of the canonical reelin pathway, whereas only full-length reelin is potent of stimulating both canonical and non-canonical reelin signaling cascade (Turk et al., 2021). Overall, current evidence implies full-length reelin to be more active than its processing-derived products (Nakano et al., 2007; Koie et al., 2014; Kohno et al., 2015; Sato et al., 2016; Dlugosz et al., 2019; Okugawa et al., 2020; Turk et al., 2021). However, it is also possible that the cleaved fragments diffuse to more distant regions to trigger downstream events (Jossin et al., 2007; Yasui et al., 2011). Reelin is processed by various enzymes at different sites of the protein and its availability therefore depends on the activity of the respective proteases (Jossin, 2020). These include ADAMTS-2, ADAMTS-3, ADAMTS-4, and ADAMTS-5, the serine protease tissue plasminogen activator, and meprin α as well as MMP-9, the latter of which triggers cleavage of reelin indirectly through the activation of ADAMTS-4 (Hisanaga et al., 2012; Krstic et al., 2012; Trotter et al., 2014; Sato et al., 2016; Ogino et al., 2017; Yamakage et al., 2019; Jossin, 2020). In contrast, the metalloproteinase inhibitors TIMP-1, TIMP-3, and α-2-macroglobulin inhibit reelin processing (Krstic et al., 2012; Jossin, 2020).

In the canonical reelin signaling pathway, reelin binds to receptors of the lipoprotein receptor family, apolipoprotein E receptor 2 (ApoER2) and very low-density lipoprotein receptor (VLDLR), and induces tyrosine phosphorylation of the intracellular adaptor protein Disabled-1 (Dab1) mediated by Src family kinases (D’Arcangelo et al., 1999; Howell et al., 1999; Arnaud et al., 2003; Förster, 2014; Jossin, 2020; Hattori and Kohno, 2021). Phosphorylated Dab1 in turn activates downstream signaling cascades, primarily influencing molecules affecting the actin and microtubule cytoskeleton but also influences adhesion molecules, ion channels, and neurotransmitter release (D’Arcangelo et al., 1999; Howell et al., 1999; Tissir and Goffinet, 2003; Jossin and Goffinet, 2007; Leemhuis and Bock, 2011; Bock and May, 2016; Ishii et al., 2016; Hirota and Nakajima, 2017; Wasser and Herz, 2017; Okugawa et al., 2020).

During cortical development, reelin is expressed by CR cells, a transient class of early-born neurons located in the MZ, the most superficial layer of the cerebral cortex (Nakajima et al., 1997; Marín-Padilla, 1998; Soriano and Del Río, 2005; Förster et al., 2006). CR cells are responsible for the proper lamination of the neocortex and hippocampus, respectively (Förster et al., 2006), by regulating the development of hippocampal connections (Del Río et al., 1997; Borrell et al., 2007), hippocampal dendrites and dendritic spines (Niu et al., 2004; Niu et al., 2008), as well as the proliferation and distribution of oligodendrocyte progenitor cells *via* the expression of reelin (Ogino et al., 2020). Furthermore, the presence and distribution of specific subtypes of CR cells during corticogenesis, as well as their timely death, appear to be important for the formation of cortical circuits (Griveau et al., 2010; Barber et al., 2015; Barber and Pierani, 2016; de Frutos et al., 2016; Causeret et al., 2021). After cortical development and demise of most CR cells, reelin then continues to be expressed primarily by interneurons in the adult brain (Alcántara et al., 1998; Drakew et al., 1998; Förster, 2014). Disruption of the reelin pathway leads to manifestation of the reeler phenotype (D’Arcangelo et al., 1995). In reeler mice, containing a genetic defect in the reelin gene, the characteristic inside-out sequence of neuronal layers in the neocortex, is reversed and neurons born later reveal a broader and irregular distribution (D’Arcangelo et al., 1995; Dekimoto et al., 2010; Boyle et al., 2011; Förster, 2014; Jossin, 2020). Reelin also regulates dendritic growth during embryonic development. For instance, dendritic growth of pyramidal cells in reeler mice (Pinto Lord and Caviness, 1979; Niu et al., 2004). The application of reelin *in vitro* has also been shown to increase dendritic growth of hippocampal neurons (Jossin and Goffinet, 2007; Matsuki et al., 2008). Conversely, a deficit of native reelin *in vivo* results in lower spine density on apical dendrites of hippocampal pyramidal neurons, which can be rescued by addition of recombinant reelin *in vitro* (Niu et al., 2008). In the developing neocortex, reelin promotes dendritic growth of pyramidal cells in early embryonic brain development (Nichols and Olson, 2010; Kupferman et al., 2014; Chai et al., 2015; Kohno et al., 2015; O'Dell et al., 2015). On the other hand, postnatally, reelin restricts dendritic growth of cortical pyramidal neurons (Chameau et al., 2009) and interneurons (Hamad et al., 2021b). As in the neocortex, reelin is also significantly involved in the proper lamination of the hippocampus (Stanfield and Cowan, 1979; Förster et al., 2006; Jossin, 2020). Thus, GCs in the DG of reeler mice do not display compact layering but are lesser in number and are diffusely distributed throughout the DG and the pyramidal cell layer is duplicated in the CA1 area (Stanfield and Cowan, 1979; Zhao et al., 2004; Förster et al., 2006; Boyle et al., 2011; Khalaf-Nazzal and Francis, 2013; Jossin, 2020). Mutants with defects in other molecules of the reelin signaling cascade display a phenotype comparable to that of the reeler mouse (Förster et al., 2006). Thus, mutants lacking the intracellular adaptor protein Dab1 or the reelin receptors VLDLR and ApoER2 show the same defects (Howell et al., 1997; Trommsdorff et al., 1999; Förster et al., 2006; Hirota et al., 2016). In contrast, mutants lacking only one of the two reelin receptors VLDLR or ApoER2 exhibit less severe migration defects and slightly different phenotypes, so that it is reasonable to assume that the two receptors exert different functions (Drakew et al., 2002; Förster et al., 2006). Findings of recent studies indicate that VLDLR suppresses neuronal invasion into the MZ, whereas ApoER2 promotes neuronal aggregation (Hirota et al., 2016; Hirota and Nakajima, 2020), speculating that the accumulation of neurons may be required for proper layer formation (Hirota and Nakajima, 2017). Mice lacking both, reelin and Dab1, have no additional defects compared to the reeler phenotype, indicating that the two proteins function in a linear pathway (Howell et al., 1999; Jossin, 2020). In the Orleans reeler strain, expression of a mutant reelin protein occurs that is not cleaved (Lambert de Rouvroit et al., 1999). Because the mutant protein is not secreted from the producing cells, it is assumed that cleavage of reelin occurs in the extracellular space (de Bergeyck et al., 1997; Hattori and Kohno, 2021). In humans, reelin deficiency leads to an autosomal recessive form of lissencephaly, characterized by abnormal neuronal lamination and lack or reduction of convolutions concomitant with cerebellar hypoplasia (Hong et al., 2000; Chang et al., 2007; Förster, 2014; Jossin, 2020). This malformation in humans is due to two different splicing mutations in the reelin gene, in both cases truncating the reelin gene, resulting in the absence of the highly basic C-terminus, which is required for normal secretion and function (D’Arcangelo et al., 1997; de Bergeyck et al., 1997; Hong et al., 2000; Manoharan et al., 2015).

## Reelin in the Context of Granule Cell Dispersion and Temporal Lobe Epilepsy

Heterozygous mutations in the reelin gene in humans may cause autosomal dominant LTLE, likely due to decreased reelin secretion (Dazzo et al., 2015; Michelucci et al., 2017; Česká et al., 2019; Michelucci et al., 2020; Dazzo and Nobile, 2021). Moreover, GABA_B_R-mediated mechanisms have been known for many years to be involved in the genesis and propagation of both typical (Crunelli and Leresche, 1991) and atypical absence seizures (Cortez et al., 2001). In this context it is interesting to note that reelin signaling has recently been shown to modulate GABA_B_ receptor function in the neocortex (Hamad et al., 2021a).

In addition, TLE may be accompanied by GCD, reminiscent of GCD in reelin-deficient reeler mice (Houser, 1990; Frotscher et al., 2003; Haas and Frotscher, 2010; Orcinha et al., 2021). However, homozygous reeler mice do not have spontaneous seizures but exhibit increased seizure susceptibility (Patrylo et al., 2006). Dab1-deficient mice also lack spontaneous seizures, but these animals display interictal epileptiform abnormalities and a considerably shortened latency to chemoconvulsant-induced SE, in which these pro-epileptogenic changes occur with decreased neurogenesis and increased numbers of ectopic GCs (Korn et al., 2016). Other studies also found that postnatal loss of Dab1 or the expression of mutated Dab1 results in ectopically placed GCs (Teixeira et al., 2012; Arimitsu et al., 2021), accompanied by decreased Dab1 phosphorylation as well as impaired synapse formation and abnormal expression of transcription factors, suggesting that the reelin-Dab1 pathway is essential for neuronal migration as well as maturation and synaptogenesis in mice (Arimitsu et al., 2021). A common hypothesis is that ectopic GCs in the hilus may contribute to the manifestation of epilepsy (Scharfman and Pierce, 2012), however, relevant studies show that mice with ectopically located GCs are largely seizure-free and misplaced GCs alone are not sufficient to evoke seizures (Koyama et al., 2012; Myers et al., 2013; Korn et al., 2016). Nevertheless, the findings of the aforementioned studies do not rule out the possibility that ectopically placed GCs may act as “hub” cells within a seizure network, as it has already been shown that these cells are hyperexcitable and make aberrant connections (Scharfman et al., 2000; Morgan and Soltesz, 2008; Zhan and Nadler, 2009; Cameron et al., 2011; Althaus et al., 2015; Korn et al., 2016). It is evident that the scattered GCs in the reeler mouse represent a developmental defect, whereas GCD in epilepsy patients could also be a secondary effect related to seizure activity (Haas and Frotscher, 2010).

In a subtype of focal cortical dysplasia (FCD) in which abnormal cortical layering occurs in association with HS and GCD, loss of reelin has been identified as the pathogenetic basis, arguing for a link between reduced reelin expression and GCD in humans (Marucci et al., 2012). Moreover, in a study performed on human hippocampal sections from MTLE patients, the extent of GCD was found to be inversely correlated with the number of reelin-expressing CR cells, suggesting a link between reelin secretion and GCD in epilepsy patients (Haas et al., 2002). In this regard, epigenetic silencing by methylation of the reelin promoter may be an underlying pathogenetic mechanism of GCD, as it was found that methylation of the reelin promoter in TLE correlated with GCD in human TLE specimens (Kobow et al., 2009). In contrast, other studies found a correlation between a high number of persistent CR cells and early complex FS (Blümcke et al., 1999) or HS (Blumcke et al., 1996). Although these findings appear contrary, they suggest a link between the reelin pathway and TLE (Blümcke et al., 2002). In addition, different staining techniques were used in the different studies for histochemical visualization of CR cells, which may also have led to the conflicting results. Nevertheless, based on the similarities between the phenotype of the reeler mouse and the pathological findings in resections of TLE patients, it seems plausible that impaired reelin signaling results in increased susceptibility to develop epilepsy. For instance, there is evidence from rodent epilepsy models, that the formation of GCD can be triggered by the loss of reelin-producing cells (Heinrich et al., 2006; Gong et al., 2007; Antonucci et al., 2008; Duveau et al., 2011; Orcinha et al., 2016). In kainate-treated rodents, GCD formation was prevented *in vivo* by infusion of exogenous reelin into the hippocampus during epileptogenesis (Müller et al., 2009). Addition of recombinant reelin to KA-treated organotypic hippocampal slice cultures could prevent GCD (Orcinha et al., 2016; Orcinha et al., 2021). Conversely, antibody blockade of reelin function in the healthy mouse hippocampus resulted in local broadening of the GCL (Heinrich et al., 2006), which could also be shown *in vitro* (Orcinha et al., 2021). While these results suggest that reelin is not only important during development, but might play an important role in maintaining the lamination of GCs in the DG of adult mice (Haas and Frotscher, 2010), recent studies using adult conditional reeler ko mice did not confirm this hypothesis, since mice with conditionally induced reelin deficiency displayed a normal neocortical and hippocampal architecture with no GCD (Lane-Donovan et al., 2015). Selective inactivation of reelin in interneurons led to subtle changes in the DG but not in the neocortex, suggesting that interneuron derived reelin does not play a major role for the layering of GCs (Pahle et al., 2020).

GCD was found to occur in the absence of neurogenesis and thus was more likely due to the displacement of differentiated neurons (Haas and Frotscher, 2010). While Mathern et al. (2002) found that neurogenesis or proliferation of neurons was not increased in the hippocampus of TLE patients (Mathern et al., 2002), others found an increase correlating with GCD (Blümcke et al., 2001; Thom et al., 2005a; Crespel et al., 2005). Again, it cannot be excluded that the use of different markers led to the contradictory results, as bromodeoxyuridine cannot be used in resected human tissue (Haas and Frotscher, 2010). The question of whether reelin deficiency, in patients without mutations in the reelin gene, is a cause or consequence of epilepsy cannot be conclusively answered in studies on patient tissue samples. Therefore, the interpretation relies on animal models. For example, in a study performed on hippocampal slice cultures it was found that differentiated neurons became motile after induction of epileptic activity by KA application, which ultimately resulted in GCD and was also associated with decreased expression of reelin as evidenced by the loss of reelin-synthesizing cells (Chai et al., 2014). This suggests, on the one hand, that epileptic activity may be a trigger for GCD and is apparently related to decreased reelin expression. In line with this, after long-term amygdala kindling, rats showed a decrease of reelin-positive neurons accompanied by ectopic GCs in the hilus of the DG (Fournier et al., 2010). Consistently, further studies showed that proteolytic processing of reelin is essential for the maintenance of GC lamination in the DG, with reelin processing found to be disrupted by epileptic conditions (Duveau et al., 2011; Tinnes et al., 2011), although reelin synthesis and secretion was normal (Tinnes et al., 2011). In this regard, epileptic states induce the upregulation of endogenous TIMP-1, thereby inhibiting matrix metalloproteinase activity, ultimately resulting in the extracellular accumulation of un-cleaved reelin (Tinnes et al., 2013). In a more recent study, the association between the occurrence of GCD and FS was investigated, and a transient increase in temperature led to the induction of GCD *in vitro*, accompanied by partial degeneration of GCs, whereas reelin-expressing CR cells were preserved in the ML, although it could not be ruled out that reelin processing was abnormal (Weninger et al., 2021). In the aforementioned publication, the appearance of GCD after temperature elevation was accompanied by severe microgliosis, suggesting an immune response of the tissue, and it was proposed that microglia could serve as markers to distinguish pathological GCD from normal variation (Roy et al., 2020; Weninger et al., 2021). Concordant with this, the results of a transcriptome signature study indicate that macrophages and microglia in particular play a critical role in epileptogenesis (Chen et al., 2020).

A previous study found a correlation between GCD and the kainate-induced loss of reelin-expressing interneurons in the DG (Orcinha et al., 2016). Thus, it was hypothesized that interneuron-derived reelin keeps the GCL compact and a decrease in reelin concentration consequently evokes migration of mature GCs toward the hilus of the DG (Haas and Frotscher, 2010; Tinnes et al., 2013; Orcinha et al., 2016). Still, selective inactivation of reelin in interneurons in conditional reelin knockout mice does not lead to GCD (Pahle et al., 2020), arguing against the importance of interneuron-derived reelin in maintaining a compact GCL.

Currently, there is only one study examining changes in the hippocampal ECM in a TLE mouse model with respect to GCD. The authors found a sequential upregulation of the ECM molecules neurocan and tenascin C at the end of the latent period in the GCL of the DG (Heck et al., 2004). After onset of the first convulsions accompanying GCD, increased, but differentially localized, expression of phosphacan as well as DSD-1 chondroitin sulfate motif and HNK-1 oligosaccharide was detected, leading the authors to conclude GCD coincides with a general increase of the ECM (Heck et al., 2004). In contrast, the expression of laminin and fibronectin was unchanged (Heck et al., 2004). Further studies of this kind could help to unravel the emergence and thus the significance of GCD in the context of epilepsy.

## Conclusion and Perspectives

All in all, the significance of GCD has not yet been clearly elucidated. Is it a pathological manifestation or the result of epileptic activity in rodents, whereas in humans it is merely a natural variation? Is it an “erroneous dogma”? Although the clinical appearance of GCD is often associated with TLE, particularly in patients with a history of FS, it has also been observed in pediatric patients without a history of FS or epilepsy. Therefore, it can be concluded that GCD is not always pathological but may also represent a non-specific naturally occurring variation. It would be important to know if this is also the case in adults, which is of course difficult to implement. Thus, for future studies, it could be helpful to differentiate disease-related GCD from normal variation. Ultimately, not only the significance of GCD, but also the relevance of FS remain controversial. The recently published large-scale study (Roy et al., 2020) has once again shown that the inclusion of adequate controls in sufficient quantities, which is difficult in the field of human epilepsy research, are of great importance for the interpretation of the results of the studies. Also, the association of FS and HS to TLE remains unclear to date, and the results of retrospective studies contrast with those of prospective studies. In addition, the role of the ECM molecule reelin in the clinical picture of TLE remains elusive. Since many studies point to a causal role of the reelin pathway in the development of GCD or TLE, molecules of this pathway could be a potential target for drug development to provide a cure for affected patients. However, the functions of reelin in the CNS are diverse, and experiments investigating the link of reelin to GCD and TLE obtained different and sometimes contradictory results. Hence, indirect mechanisms should also be considered, as reelin spins a complex network with a variety of different ECM molecules, influencing each other. An example is provided by MMP-9, known to be upregulated by epileptic activity and being able to process reelin indirectly *via* ADAMTS-4 as already described. Altogether, many questions remain unanswered on this topic and further studies, both on animal models and human tissue, will be needed to ultimately answer them.
